# Academic Interest Scale for Adolescents: Development, Validation, and Measurement Invariance With Chinese Students

**DOI:** 10.3389/fpsyg.2019.02301

**Published:** 2019-10-11

**Authors:** Zheng Luo, Yun Dang, Wenjie Xu

**Affiliations:** Beijing Key Laboratory of Learning and Cognition, School of Psychology, Capital Normal University, Beijing, China

**Keywords:** Academic Interest Scale for Adolescents (AISA), academic interest, four-phase interest development model, scale development, measure invariance

## Abstract

Hidi and Renninger’s four-phase interest development model was identified as the most complete and widely used theoretical model illustrating the essence of academic interest. Using the model along with current research literature as a basis, this study aimed to develop and initially validate a generic multidimensional instrument to measure academic interest across different school subjects in the Chinese education context; this instrument was called the Academic Interest Scale for Adolescents (AISA). Three large samples of Chinese junior high school students were recruited by cluster sampling in the study. (1) Sample 1 (*N* = 552; 45.5% girls; 12.31 [*SD* = 0.98] years, range = 10–15 years) completed the draft of AISA, Intrinsic Motivation Scale and Scale for Adolescents’ Flow State in Learning in math and English. (2) Sample 2 (a subgroup of Sample 1, 411 students) completed the AISA in math and English again 2 months later after the first survey. (3) Sample 3 (*N* = 1,780; 50.1% girls; 13.69 [*SD* = 0.97] years, range = 12–16 years) completed the AISA in math, English, and Chinese. Identically worded items were used in AISA, except for the name of the subject. An exploratory factor analysis for math in sample 1 using principle axis factoring and promax rotation resulted in a 29-item AISA containing four dimensions: emotion, value, knowledge, and engagement, and the latent variables together explained 59.40% of the total variance. Confirmatory factor analysis for math, English, and Chinese in sample 3 suggested the four-factor model fits well in different samples and subjects. Scale scores showed adequate internal consistency (the Cronbach’s α for AISA and each subscale ranged from 0.86 to 0.93) and acceptable test-criterion relationships (correlations between the AISA score and intrinsic motivation and flow state in learning > 0.51, *ps* < 0.001). Furthermore, the structural measure invariance across subjects, time (2-month interval), genders and grades were upheld. The AISA promises to be a useful tool for the evaluation of academic interest among Chinese adolescents and can be administered in different educational settings, i.e., different subjects, time, genders, and grades.

## Introduction

Since Herbart (1776–1841) began to consider fostering interest as one of the primary goals of education, researchers have investigated the contribution of student interest to academic achievement. It has been widely acknowledged that interest promotes engagement, efficiency, effort, and persistence in learning (e.g., Dewey, 1913; Mitchell, 1993; Lipstein and Renninger, 2006; Trautwein et al., 2015). Unfortunately, studies have indicated that interest in most school subjects shows a downward trend over time (Hidi and Harackiewicz, 2000; Krapp, 2002; Dotterer et al., 2009; Frenzel et al., 2010). This trend not only occurs in primary school but also seems to be more obvious in middle school (Prenzel, 1998). In China, the lack of academic interest is common among primary and middle school students. There is even a term for it known as learning weariness. A Study found that 17.38% junior high school students in rural areas of China reported to have suffered from learning weariness (Zhao, 2015). Theoretical as well as empirical research emphasized that students’ individual factors (e.g., Schurtz et al., 2014; Zhang et al., 2018; Denner et al., 2019) along with environmental factors (e.g., Lazarides et al., 2019) are all important predictors of their academic interests. As for learning weariness of Chinese students, one of the main reasons is the heavy academic burden (Guo and Zhang, 2012).

A number of self-report instruments have been developed to assess the level of academic interest and associations with academic performance among adolescents in Western countries (e.g., Dotterer et al., 2009; Kalender and Berberoglu, 2009; Linnenbrink-Garcia et al., 2010; Rotgans, 2015). Most of these scales were limited to measurement of emotion or value components in academic interest. Meanwhile, the measurement invariance of the instrument across subjects, time, genders, and grades has not been fully tested. In China, most early academic interest measures adopted single item or single dimension, which have not yet been fully validated (Tu and He, 2013). The current measures focus on subject-specific interest, such as mathematics learning interest questionnaire (MLIQ, Wu and Liu, 2017) for junior high school students and sports learning interest scale (SLIS, Lin and Chai, 2017) for primary and secondary school students. Therefore, to study the multifactorial nature and academic effects of academic interest and provide effective prevention and intervention ideas for loss of interest, it is necessary to develop a more comprehensive tool for assessing Chinese adolescents’ academic interest across different educational contexts.

### Definition of Academic Interest

Interest, which is a unique motivational variable, refers to a preferred engagement of a person with a specific object (i.e., a certain topic, activity, and idea), which can display itself as a psychological state as well as the relatively enduring predisposition toward these objects (Hidi and Renninger, 2006). According to the person-object theory of interest (POI; Krapp, 2000, 2005), the development of interest relies on the ongoing interactions between the environment (object) and the person. Furthermore, interest can be divided into situational interest and individual interest (e.g., Hidi, 1990; Krapp et al., 1992; Krapp, 2000, 2005). Situational interest is a state of focused attention and affective reaction elicited by current environmental stimuli (Hidi and Baird, 1986; Hidi, 1990), Whereas individual interest is a sustained preference for particular content (Krapp and Fink, 1992; Renninger, 2000). Individual interest develops from situational interest (Hidi and Renninger, 2006). Both types of interest have been shown to positively influence attention, cognitive performance, and affection (Hidi, 1990), although individual interest tends to have more enduring effects. Descriptors of interest in learning have included a host of names (e.g., academic, individual, personal, cognitive), which have been used interchangeably to some extent. Earlier research tended to focus on general interest across subjects. However, students often are more interested in some school subjects than in others. Contemporary research has stressed that interest is a domain-specific construct (e.g., Gogol et al., 2017), and that it is necessary to measure interest separately for different school subjects (e.g., math interest, English interest, etc.). The concept of academic interest to which this paper refers represents the individual interest of adolescents related to school tasks, focusing on subject-specific or subject interest.

### Four-Phase Interest Development Model and Constituents of Academic Interest

Hidi and Renninger (2006) developed a four-phase interest development model based on POI, which is the most complete and widely used model illustrating the essence of academic interest. The first two phases, *triggered situational interest* and *maintained situational interest*, are included under *situational interest*, which is sparked by environmental stimuli and temporary sustained by support from others. The last two phases, *emerging individual interest* and *well-developed individual interest*, belong to *individual interest*, the motivation of which results mainly from individuals themselves and partially from the support of others (Hidi and Renninger, 2006).

Hidi and Renninger (2006) suggested that “each phase of interest is characterized by varying amounts of affect, knowledge, and value.” Furthermore, engagement in learning activities differs in each phase. Well-developed individual interest promotes self-regulation and enthusiasm to engage and reengage in learning activities, leading to the individual’s persistence when confronted with difficult situations (Hidi and Renninger, 2006). We can, therefore, further divide academic (individual) interest into four components: *emotion*, *value*, *knowledge*, and *engagement*. The component of emotion refers to the positive feelings accompanying the activities, such as pleasure, excitement and enjoyment (Izard, 1991; Schiefele, 1991; Csikszentmihalyi and Hunter, 2003; Hidi and Renninger, 2006). The component of value refers to the perception of the personal significance of the specific object or domain, such as its importance to individual development (Schiefele, 1991; Krapp, 1999; Hidi and Renninger, 2006). The component of knowledge refers to the perception of stored knowledge in a specific domain. Krapp (2000) suggested that stored knowledge can be used to assess interest due to the positive correlations between them (Alexander and Jetton, 1994). The component of engagement refers to the predisposition to participate in specific learning activities. Students who have a high level of individual interest prefer to join in more learning activities (Schiefele, 1991; Tobias, 1994; Mazer, 2013). Meanwhile, engagement can facilitate the sustaining and deepening of interest for specific object or content (e.g., Csikszentmihalyi and Rathunde, 1992; Renninger, 2000; Hidi et al., 2004).

### Potential Limitations of the Existing Academic Interest Instruments

A closer examination of the existing academic interest measures in the Western contexts and Asian has revealed a number of limitations that this study aims to address.

First, there is a need to further develop academic interest scales for adolescents based on the four-phase interest development model. In accordance with different conceptualizations and operationalizations of interest, a variety of academic interest instruments have been used across studies, which involved single, two- or three-components measures. Single component measures use emotion or value as the only indicator of academic interest measures with one or more items (Nurmi and Aunola, 2005; Dotterer et al., 2009; Kalender and Berberoglu, 2009; Viljaranta et al., 2014; Jõgi et al., 2015). Two-component measures included both emotion and value, such as the Study Interest Questionnaire (SIQ, Schiefele et al., 1988) and academic interest scales developed by Linnenbrink-Garcia et al. (2010), Maurice et al. (2014), and Høgheim and Reber (2015). Only a few three-component measurements have been developed. In addition to emotion and value, the other component may be knowledge or engagement. For example, the General Individual Interest Scale (GIIS), developed by Tang and Toyama (2016) in Japan, is divided into three subscales (emotion, value, and knowledge) to assess undergraduates’ academic interest. Rotgans (2015) developed an individual interest scale for high school students in Singapore. The seven-item scale assessed three aspects of academic interest: positive emotion, value, and predisposition to reengage with particular content.

The four-phase interest development model provides a broader and more comprehensive theoretical framework for the measurement of interest. As mentioned earlier, the construct of academic interest is multidimensional according to the model, including the components of emotion, value, knowledge, and engagement. Four-component measures of academic interest are rare. To the best of our knowledge, only one scale for the subject of math, which used a sample of elementary students, has been developed to date in the U.S. (Wininger et al., 2014). Two Chinese scales, MLIQ and SLIS, adopted the four-phase interest development model. One problem for the two scales is that their dimensions are different phases. MLIQ comprises three dimensions: triggered situational interest, maintained situational interest, and individual interest. SLIS comprises four dimensions: triggered situational interest, maintained situational interest, emerging individual interest and well-developed individual interest. Their dimensions overlap to some extent. For example, emotion may be included in different subscales. Therefore, the development of a new scale for Chinese adolescents based on the essence of academic interest according to four-phase interest development model is justified.

Second, there is a need to further develop interest measures for adolescents that can be generally used in different subjects. Most researchers have held that academic interest differed across subjects (e.g., Schiefele, 1991; Krapp, 2002; Hidi and Renninger, 2006). For example, students hold different levels of interest in biology, chemistry, and physics (Jansen et al., 2016). Students with higher interest in French were found to have a lower interest in German (Gogol et al., 2015). Some subject-specific academic interest scales have assessed interest only in a particular narrowly defined subject, such as math (e.g., Wininger et al., 2014). MLIQ and SLIS are all subject-specific interest scales. MLIQ consists of 17 items such as “I like to inquiry the in and out of mathematical principles and formulas” to assess math interest (Wu and Liu, 2017). SLIS includes items like “I might imitate the actions of my favorite athletes” to assess sports learning interest. As can be seen from the items, it is difficult for other researchers to use these instruments in different domains or subjects. Meanwhile, some empirical research has replaced the name of the domain in items to directly measure certain interest (e.g., Nurmi et al., 2011; Jansen et al., 2016). This approach ignores whether academic interests have the same structure among different subjects. To be able to work across subjects to compare academic interest, parallel scales with equivalent measurement structures and items to measure academic interest in multiple academic subjects for each person are required. Nonetheless, few questionnaires currently exist that have been developed based on this premise. Moreover, to be able to test intraindividual and interindividual differences across subject domains, measurement instruments need to reflect strong measurement invariance across subjects (Meredith, 1993), i.e., scores in one subject need to be comparable to scores in another subject. As far as we know, only one study has been reported in the literature testing measurement invariance across subjects. Rotgans (2015) established the metric invariance of the one-factor interest model across three subjects (chemistry, geography, and history) using a multi-group comparison approach.

Third, there is a need to further test measurement invariance across time, genders, and grades in academic interest instruments. Academic interests are often compared across time. For example, longitudinal research has shown that students’ academic interest declines over time (Dotterer et al., 2009; Frenzel et al., 2010). Students’ academic interest in math, physics and chemistry subject obviously declines over time most, but their interest in biology has not been shown to decline significantly (Todt et al., 1998). For subjects in science domains, girls’ interest declines significantly faster than boys (Hoffmann, 2002). Additionally, gender and grade differences on the mean level in academic interest have also been documented (Koller et al., 2001; Frenzel et al., 2010). Measurement invariance is a prerequisite for comparing these differences. To be able to compare latent means across time, genders and grades, factor loadings and item intercepts must be invariant (Meredith, 1993). If invariance is not given, the differences in observed test scores in different groups or at different times does not necessarily reflect true differences. However, measurement invariance of such academic interest scales across time, genders, and grades has been seldom tested. The invariance of the academic interest instruments has not yet been tested in China, as far as we know.

Based on the above, our first objective of the present study was to develop the Academic Interest Scale for Adolescents (AISA) for assessing multiple academic interest facets across school subjects among adolescents in the Chinese education context. Through creating items suitable for different subjects, expert feedback, cognitive interview, exploratory factor analysis (EFA) and confirmatory factor analysis (CFA), we expected to obtain a generic academic interest scale for Chinese adolescents with four-factor latent structure. Our second objective was to preliminarily validate the AISA. We expected to observe adequate internal consistency and satisfactory test-criterion relationships. Our third objective was to test the measure invariance across subjects, time, genders and grades, and expected to observe invariances in measurement and structure.

## Materials and Methods

### Design

This study consisted of three cross-sectional questionnaire surveys in three large samples of Chinese junior high school students recruited by cluster sampling. The goal of the first survey was to develop and initially validate the AISA scale in two subjects (math, English). The second survey was to test measurement invariance across time. The third survey was intended to further validate the factorial structure spanning across three subjects (math, English, and Chinese), and tested the measurement invariance across subjects, genders, and grades. The first survey was conducted in September 2017, the second was in November 2017, and the third was in October and November 2018.

### Participants

#### Sample 1

A total of 552 students (45.5% girls; age: *M* = 12.31 years, *SD* = 0.98, range = 10–15 years) were recruited from 12 classrooms in grades 6–8 in two public junior high schools of central China’s Henan Province. The only child rate was 29%. Paternal and maternal education levels were 2.5 and 0.9% for graduate school and above, 29.7 and 25.7% for university, 34.4 and 33.9% for senior high school, and 33.5 and 39.5% for junior high school and below, respectively. The number of participants in each grade and their gender is shown in Table 1. Among these, 552 participants completed the AISA scale, Intrinsic Motivation Scale (IMS) and Scale for Adolescents’ Flow State in Learning (SAFSL) in math and 525 participants completed the same scales in English.

**TABLE 1:** The number of participants in each grade as well as their gender.

**Subject**	**Gender**	**Grade 6**	**Grade 7**	**Grade 8**	**Grade 9**	**Total**
Sample 1	Boys	101	99	101	–	301
	Girls	105	76	70	–	251
	Total	206	175	171	–	552
Sample 3	Boys	–	372	330	186	888
	Girls	–	337	312	243	892
	Total	–	709	642	429	1780

#### Sample 2

Two months later, a subgroup of 411 students for math and 396 students for English who participated in the first survey completed the AISA in math and English again. The demographic characteristics were similar to those of the first sample. The data was used to assess measure invariance over time.

#### Sample 3

A total of 1,780 students (50.1% girls; age: *M* = 13.69 years, *SD* = 0.97, range = 12–16 years) were recruited from 48 classrooms in grades 7–9 in 13 junior high schools of Beijing, China. The only child rate was 62.3%. Paternal and maternal education levels were 6.9 and 5.3% for graduate school and above, 29.9 and 30.8% for university, 38.9 and 40.4% for senior high school, 23.3 and 22.0% for junior high school and below, respectively; 1.0% of the paternal educational level and 1.5% of the maternal educational level were unknown. All participants completed the AISA scale in math, English, and Chinese. The number of participants in each grade as well as their gender is shown in Table 1.

The experimental protocol and anonymous informed consent were approved by the Ethics Research Committee (ERC) of School of Psychology, Capital Normal University, to protect participants’ privacy. Written approval for the research was provided by the principal of each participating school before the data collection occurred. Students participated in the research voluntarily and provided anonymous informed verbal assent. The parents were fully informed of the research purpose and procedure and provided their anonymous verbal informed consents to have their children participate. Written informed consent from the parents was not obtained because the ERC waived this requirement to protect students’ anonymity and privacy, as the consent document could link the participant to the research.

### Development of Academic Interest Scale for Adolescents (AISA)

#### Item Creation

The first step in scale construction was to develop linguistic definitions of academic interest and the scope of contents according to the four-phase interest development model through group discussion. The AISA item pool was drawn from the theoretical rationale described above and existing measures of individual interest (e.g., Harackiewicz et al., 2008; Schiefele, 2009; Linnenbrink-Garcia et al., 2010; Wininger et al., 2014; Rotgans, 2015; Tang and Toyama, 2016; Wang and Adesope, 2016; Wu and Liu, 2017). We included as many items as possible in the item pool that were suitable for different subjects for further item/factor selection. A total of 81 items including four domains were initially generated: 23 items for emotion (e.g., “I enjoy studying …”), 20 items for value (e.g., “The knowledge of … promotes my growth”), 18 items for knowledge (e.g., “I know all kinds of things about …”), and 20 items for engagement (e.g., “I want to learn things that are not included in … textbooks”). All items were scored on a five-point Likert scale: 1 represented “strongly disagree,” and 5 represented “strongly agree.” We used these items with identical wording (except for the name of the subject) to assess individual interest in math, English and Chinese, replacing “…” with “math” or “English” or “Chinese.”

#### Expert Feedback

Seven experts, including five researchers on educational psychology and two junior high school teachers, were invited to review the items. The experts received our definition of academic interest and a rationale for scale development. They were asked first to rate whether each item clearly assessed defined content using a five-point Likert scale ranging from strongly disagree to strongly agree. Means and standard deviations were calculated. All items ranked above three and were kept. The experts were also asked to modify the items if they thought there was something inappropriate (e.g., not easy to understand; not fit for different school subjects) and add new items to include the entire scope of academic interest based on their experience. Two authors revised the items according to the experts’ suggestions. For example, in Item 37, the word “relaxed” was replaced with “excited.” Fifteen items were amended, and no new items were added.

#### Cognitive Interview

Cognitive interviews examining the 81 items in the draft were conducted with a convenience sample of six junior high school students. The items were divided into three approximately equal parts. Each participant completed one part, and each part was completed by two participants. Three graduate research assistants administered items to participants, asked them to explain their understanding of each item and corresponding response options, and noted items that participants perceived as confusing or unclear. Interviews lasted between 40 and 60 min and were audio-recorded. Based on feedback obtained from the interviews, minor wording changes were made to four items. As a result, a total of 81 items were generated for pilot study.

### Other Instruments

#### Intrinsic Motivation Scale (IMS)

Served as one of the criterion measures, the IMS (Elliot and Church, 1997; Wang et al., 2006) was used to assess participants’ intrinsic motivation toward their math/English class. This scale includes eight items using a five-point Likert scale (from *strongly disagree* to *strongly agree*). Internal consistency in both subjects (composite reliability = 0.88 and 0.85) was acceptable in this study.

#### Scale for Adolescents’ Flow State in Learning (SAFSL)

Served as one of the criterion measures, the SAFSL (Lei et al., 2012) was used to assess participants’ flow state when they become engaged in math/English learning. This scale includes 12 items on four dimensions (definite goals of learning, concentration on task and enjoyment, loss of self-consciousness and distortion of time perception). Participants used 1 (*never happened*) to 5 (*always happened*) scales to indicate their responses. Internal consistency for the entire scale and subscales in both subjects were adequate in this study. For the entire scale, the composite reliability was 0.95 in math and 0.96 in English. For each of the subscales the composite reliability was 0.76, 0.87, 0.82, 0.73 respectively in math, and 0.86, 0.92, 0.86, 0.77 respectively in English.

### Statistical Analyses

The data analysis was performed with Mplus 7.0 (Muthén and Muthén, 2012). The percentage of missing data among items was 1.2% in sample 1, 1.0% in sample 2, and 0.3% in sample 3. Little’s MCAR test was conducted, and all the missing data in the different subjects of the three samples were confirmed to be missing completely at random (387.21 ≤ χ^2^s ≤ 9505.94, 456 ≤ *df*s ≤ 9545, *p*s ≥ 0.47). Meanwhile, the mean values of all items ranged from 1.62 to 4.46. The standard deviations ranged from 0.79 to 1.35. The skew and kurtosis indices ranged from −1.55 to 1.79 and from −1.12 to 2.42, respectively. Following Kline’s (2005) recommendations, the data in this study were considered to be univariate normal. We used the full-information maximum likelihood estimation (FLML) implemented in Mplus 7.0 to address missing values, which utilize all available information when estimating the model parameters (Schafer and Graham, 2002).

An EFA for math in sample 1 was conducted to refine the scale and determine the factorial structure using principle axis factoring and promax rotation. CFAs for math, English, and Chinese in sample 3 were conducted separately to examine the factorial structure of the scale using maximum likelihood (ML). We assessed the model fit using multiple indices: the comparative fit index (CFI), the Tucker-Lewis index (TLI), the root mean square error of approximation (RMSEA), and the standardized root mean square residual (SRMR). CFI and TLI values greater than 0.90 (Hu and Bentler, 1999), SRMR values less than 0.08 (Hu and Bentler, 1999), and RMSEA values less than 0.08 (Mcdonald and Ho, 2002) are considered to indicate adequate model fits to the data, respectively. However, it should be noted that these criteria are arbitrary (Hu and Bentler, 1999; Hau et al., 2004). Additionally, chi-square to the degrees of freedom (χ^2^/df) values less than 5 are considered to indicate an excellent model fit (Kline, 2005). Given the actually large sample size, fit should not be over-interpreted.

The internal consistency for the AISA and each of the subscale was assessed using composite reliability and the Cronbach’s α in sample 1. Composite reliability and the Cronbach’s α greater than 0.70 indicate adequate homogeneity (Nunnally and Bernstein, 1994). The test-criterion relationships were assessed using bivariate correlations between academic interest evaluated by the AISA and intrinsic motivation and flow state in learning in sample 1.

Tests of measurement invariance were used to determine whether the measurement structure is consistent across time (sample 2) and across subjects, genders, and grades (sample 3). We conducted the tests across academic subjects by specifying the latent factors in different subjects as separate factors and tests across time, subjects, genders, and grades by using multiple-group analyses within the CFA framework. In accordance with the steps outlined by Meredith (1993) and Vandenberg and Lance (2000), a series of nested models with increasing invariance restrict were conducted. Before these tests, we reported the goodness of fit for the models separately in different subjects, time, genders, and grades to ensure each of them was reasonable. Then, four models were performed successively. Model 1 was a configural invariance model with identical loading patterns but no invariance for any parameters. Model 2 was a metric invariance (weak invariance) model with factor loadings constrained to invariant across subjects, time, genders, and grades. Model 3 was a scale invariance (strong invariance) model with additionally constrained item intercepts to be equality across subjects, time, genders, and grades. Model 4 was a structural invariance model with factor variances and covariances (in addition to invariant factor loadings and item intercepts) constrained to equality across subjects, time, gender, and grades.

The fit of the constrained model and the unconstrained model were compared in terms of their χ^2^ values. A non-significant increase in the χ*^2^* value (relative to *df*) in the constrained model compared to the unconstrained model indicated that the constrains across groups were possible. As an additional criterion, the change in the CFI coefficient was considered. If the decrease in CFI value of the constrained model compared to the unconstrained model was more than 0.01 (ΔCFI < −0.01), the constrained model was not supported, which indicate a lack of invariance. The ΔCFI criterion was argued to be superior to Δχ*^2^*, as it is less sensitive to sample size (Cheung and Rensvold, 2002). Configural invariance means that the pattern of factors is equivalent across subjects, time, genders, and grades (Horn and Mcardle, 1992). Metric invariance (invariant factor loadings) implies equality of scaling units across subjects, time, genders, and grades (Cole and Maxwell, 1985; Marsh, 1994). Scalar invariance (invariant intercepts) implies that intercepts of items’ regressions on the factor are invariant across subjects, time, genders, and grades (Meredith, 1993). Finally, structural invariance (invariant factor variance and covariances) represents that differences in factor variances and covariances are interpreted as reflecting differences in the calibration of true scores and in conceptual associations among the true scores across subjects, time, genders, and grades (Schmitt, 1982).

## Results

### Exploratory Factor Analysis (EFA)

Because math is a main subject for Chinese students and the AISA for math was implemented in all three surveys, we first conducted an EFA using principle axis factoring and promax rotation on the 81 items for math in sample 1 to determine the factorial structure of the AISA scale. We used parallel analysis (Horn, 1965) to determine the number of factors to retain, which is considered a more accurate criterion compared with eigenvalues greater than 1 and the scree test (Hayton et al., 2004). Fifty randomly generated simulated data sets indicated 95% confidence intervals (CIs) of 1.851–1.874, 1.781–1.796, 1.705–1.758, 1.700–1.712, and 1.665–1.674 for the eigenvalue of the first five random factors, respectively. In the actual data set, only the factors with eigenvalues greater than the upper limit of these CIs would be retained. A four-factor model was supported.

Four items with item-total correlations of less than 0.30 were removed after item analysis. Furthermore, all items with loadings of 0.30 and lower were also removed from further analysis (Nunnally and Bernstein, 1994). Ultimately, 52 items in math models were excluded based on lower factor loadings, crossed loadings (loading greater than 0.30 in two or more factors), and item analysis statistics. A 29-item scale was generated. The eigenvalues of the four factors were 15.093, 1.763, 1.158, and 1.013, respectively. The latent variables explained 13.29, 10.56, 9.45, and 7.30% of variance, respectively, and together explained 59.40% of the total variance. Interfactor correlations ranged from 0.59 to 0.67. The factors were labeled as emotion, value, knowledge, and engagement. Specifically, the emotion, value and engagement subscales consisted of seven items each, whereas the knowledge subscale included eight items. The final items in English and Chinese are described in the Appendix. Factor loadings and item-total correlations can be seen in Table 2.

**TABLE 2:** Rotated factor loadings in the EFA and item-total correlation for math in sample 1 (N_math–1_ = 552) and factor loadings for subjects in sample 3 (N_3_ = 1,780) in the CFA.

	**EFA**				**Item-total** **correlation**	**CFA**		
**Item**	**1**	**2**	**3**	**4**		**Math-3**	**English-3**	**Chinese-3**
3	**0.675**	0.134	0.052	0.080	0.806	0.760	0.746	0.741
5	**0.592**	0.037	0.146	0.163	0.806	0.797	0.800	0.755
8	**0.673**	0.090	0.122	0.058	0.808	0.796	0.807	0.793
13	**0.542**	0.101	0.050	0.215	0.782	0.797	0.785	0.762
14	**0.622**	0.084	0.096	0.142	0.808	0.806	0.825	0.811
18	**0.561**	0.111	0.196	0.045	0.788	0.774	0.799	0.793
26	**0.687**	0.095	0.067	0.103	0.817	0.805	0.785	0.742
1	−0.012	**0.648**	0.165	−0.117	0.593	0.582	0.636	0.573
10	0.033	**0.488**	0.140	0.056	0.629	0.606	0.656	0.629
16	−0.048	**0.728**	−0.058	0.165	0.675	0.698	0.721	0.670
17	0.046	**0.504**	0.016	0.174	0.645	0.740	0.753	0.732
20	−0.001	**0.602**	0.218	0.003	0.708	0.782	0.793	0.794
24	0.105	**0.799**	−0.092	0.015	0.705	0.769	0.789	0.769
27	0.037	**0.804**	−0.031	−0.001	0.688	0.814	0.819	0.802
29	0.052	**0.745**	0.005	0.010	0.695	0.803	0.798	0.786
4	0.042	0.036	**0.561**	0.054	0.615	0.652	0.681	0.616
7	0.299	0.032	**0.612**	−0.163	0.686	0.750	0.765	0.686
9	0.174	0.025	**0.569**	0.043	0.704	0.761	0.763	0.680
12	0.120	0.071	**0.422**	0.265	0.758	0.782	0.781	0.742
15	0.118	−0.036	**0.510**	0.145	0.646	0.710	0.716	0.675
22	−0.042	−0.025	**0.648**	0.195	0.674	0.733	0.757	0.725
23	−0.115	−0.006	**0.767**	0.111	0.657	0.752	0.759	0.719
2	−0.018	0.020	0.072	**0.620**	0.617	0.492	0.546	0.464
6	0.264	0.181	0.003	**0.484**	0.800	0.708	0.761	0.715
11	0.043	−0.059	0.190	**0.621**	0.696	0.659	0.725	0.664
19	0.155	0.106	0.019	**0.607**	0.763	0.787	0.782	0.793
21	−0.014	−0.005	0.133	**0.398**	0.480	0.517	0.609	0.529
25	0.033	0.237	0.092	**0.309**	0.594	0.633	0.615	0.582
28	0.230	0.168	0.115	**0.357**	0.756	0.742	0.757	0.736

*EFA, exploratory factor analysis; CFA, confirmatory factor analysis. The bold values mean items with a strong loading (0.30 or higher) on one factor.*

### Confirmatory Factor Analyses (CFAs)

To further verify the factorial structure of the 29-item AISA, CFAs for four-factor models in math, English, and Chinese subjects (sample 3) were computed separately. Model fits were all acceptable (Table 3). In sample 3, all factor loadings of math ranged from 0.492 to 0.814. For English, factor loadings ranged from 0.546 to 0.825. For Chinese, factor loadings ranged from 0.464 to 0.811 (Table 2).

**TABLE 3:** Model fit statistics for CFA in sample 3 (*N* = 1,780).

**Model**	**χ*^2^***	***df***	**CFI**	**TLI**	**SRMR**	**RMSEA**	**Total of**
						**(90%CI)**	**items**
Math-3	3205.109	371	0.913	0.905	0.041	0.066 (0.063, 0.068)	29
English-3	2890.026	371	0.927	0.920	0.037	0.062 (0.060, 0.064)	29
Chinese-3	3058.911	371	0.910	0.901	0.041	0.064 (0.062, 0.066)	29

*χ*^2^*, chi-square; *df*, the degrees of freedom; CFI, comparative fit index; TLI, Tucker-Lewis index; SRMR, standardized root mean square residual; RMSEA, root mean square error of approximation; CI, confidence interval.*

### Tests of the Internal Consistency

Based on the data from the first survey (N_math–__1_ = 552; N_English–__1_ = 525), the composite reliability (CR) and Cronbach’s alpha values indicated that the AISA and each subscale were internally consistent. For the total AISA, the CR was 0.98 and α was 0.80 in math, the CR was 0.98 and α was 0.83 in English. For the emotion, value, knowledge, and engagement subscale, the CR was 0.80, 0.91, 0.88, 0.86 and α was 0.87, 0.90, 0.88, 0.86 in math, the CR was 0.80, 0.93, 0.93, 0.91 and α was 0.90, 0.93, 0.93, 0.91 in English.

### Tests of Test-Criterion Relationships

Within the sample from the first survey (N_math–__1_ = 552; N_English–__1_ = 525), bivariate correlations indicated satisfactory test-criterion relationships between the AISA and intrinsic motivation and flow state in learning, respectively. For math, the AISA total score had strong, significant positive correlations with the intrinsic motivation and the flow state in learning, correlations coefficients were 0.95 and 0.87 (*ps* < 0.001). For English, the AISA total score had moderate, significant positive correlations with the intrinsic motivation and the flow state in learning, correlations coefficients were 0.51 and 0.60 (*ps* < 0.001).

### Tests of Measurement Invariance Across Academic Subjects

Using the data from sample 3, we examined four levels of invariance (configural invariance, metric invariance, scalar invariance, and structural invariance) between math, English, and Chinese subject. As shown in Table 4, configural invariance with no further constraints was supported by fit indices meeting benchmarks for adequate fit, χ^2^/*df* = 8.225, CFI = 0.917, TLI = 0.909, SRMR = 0.039, RMSEA = 0.064 (0.063, 0.065). Metric, scalar, and structural invariance could be assumed between math and English subjects, as evidenced by a non-significant drop in model fit (ΔCFI > −0.01) for the successively stricter models.

**TABLE 4:** Model fit statistics for models representing different degrees of invariance across academic subjects (*N* = 1,780).

**Model**	**χ*^2^***	***df***	**Δχ*^2^***	**Δ*df***	**CFI**	**TLI**	**SRMR**	**RMSEA (90%CI)**	**ΔCFI**
Math	3205.11	371	—	—	0.913	0.905	0.041	0.066 (0.063, 0.068)	
English	2890.03	371	—	—	0.927	0.920	0.037	0.062 (0.060, 0.064)	
Chinese	3058.91	371	—	—	0.910	0.901	0.041	0.064 (0.062, 0.066)	
Configural invariance	9154.045	1113	—	—	0.917	0.909	0.039	0.064 (0.063, 0.065)	
Metric invariance	9233.031	1163	78.986^∗∗^	50	0.917	0.913	0.042	0.062 (0.061, 0.064)	0.000
Scalar invariance	9789.602	1213	556.571^∗∗∗^	50	0.912	0.911	0.045	0.063 (0.062, 0.064)	–0.005
Structural invariance	9844.775	1233	55.173^∗∗∗^	20	0.911	0.912	0.059	0.063 (0.061, 0.064)	–0.001

*Δχ*^2^* = difference in χ*^2^* value of the constrained model compared to the unconstrained model. Δ*df*, difference in *df* value of the constrained model compared to the unconstrained model. ΔCFI, difference in CFI value of the constrained model compared to the unconstrained model. ^∗∗^*p* < 0.01, ^∗∗∗^*p* < 0.001.*

### Tests of Measurement Invariance Over Time

As a requirement for comparison over time, we examined four levels of invariance (configural invariance, metric invariance, scalar invariance, and structural invariance) over time in math and English subject separately in sample 2. The test of the configural invariance model with no further constraints resulted in an adequate fit to the data in two subjects, for math, χ*^2^/df* = 1.944, CFI = 0.918, TLI = 0.912, SRMR = 0.038, RMSEA = 0.048 (0.045, 0.050); for English, χ*^2^/df* = 2.019, CFI = 0.926, TLI = 0.920, SRMR = 0.039, RMSEA = 0.051(0.048, 0.053). The models for test metric, scalar, and structural invariance had no substantial changes in model fit (ΔCFI > −0.01), indicating that metric, scalar, and structural invariance held over time for both academic subjects (Table 5).

**TABLE 5:** Model fit statistics for models representing different degrees of invariance over time (N_math_ = 411; N_English_ = 396).

**Model**	**χ^2^**	***df***	**Δχ*^2^***	**Δ*df***	**CFI**	**TLI**	**SRMR**	**RMSEA (90%CI)**	**ΔCFI**
**Math**									
Time 1	772.700	371	—	—	0.948	0.943	0.037	0.051 (0.046, 0.056)	
Time 2	1139.448	371	—	—	0.916	0.908	0.041	0.071 (0.066, 0.076)	
Configural invariance	2989.698	1538	—	—	0.918	0.912	0.038	0.048 (0.045, 0.050)	
Metric invariance	3026.801	1563	37.103	25	0.917	0.913	0.042	0.048 (0.045, 0.050)	−0.001
Scalar invariance	3130.287	1588	103.48^∗∗∗^	25	0.913	0.909	0.043	0.049 (0.046, 0.051)	−0.004
Structural invariance	3159.186	1598	28.899^∗∗^	10	0.912	0.909	0.045	0.049 (0.046, 0.051)	−0.001
**English**									
Time 1	1014.022	371	—	—	0.934	0.928	0.041	0.066 (0.061, 0.071)	
Time 2	1070.683	371	—	—	0.933	0.926	0.037	0.069 (0.064, 0.074)	
Configural invariance	3105.249	1538	—	—	0.926	0.920	0.039	0.051 (0.048, 0.053)	
Metric invariance	3144.131	1563	38.882^∗^	25	0.925	0.921	0.043	0.051 (0.048, 0.053)	−0.001
Scalar invariance	3244.763	1588	100.632^∗∗∗^	25	0.922	0.918	0.045	0.051 (0.049, 0.054)	−0.003
Structural invariance	3262.186	1598	17.423	10	0.921	0.919	0.046	0.051 (0.049, 0.054)	−0.001

*^∗^*p* < 0.05, ^∗∗^*p* < 0.01, and ^∗∗∗^*p* < 0.001.*

### Tests of Measurement Invariance Across Genders

We examined measurement invariance across students’ genders for the three subjects separately in sample 3. As shown in Table 6, the test of the configural invariance model with no further constraints was supported in three subjects, for math, χ^2^/*df* = 4.900, CFI = 0.912, TLI = 0.904, SRMR = 0.044, RMSEA = 0.066 (0.064, 0.068); for English, χ^2^/*df* = 4.565, CFI = 0.922, TLI = 0.915, SRMR = 0.040, RMSEA = 0.063 (0.061, 0.065); for Chinese, χ^2^/*df* = 4.583, CFI = 0.911, TLI = 0.902, SRMR = 0.043, RMSEA = 0.063 (0.061, 0.066). Metric, scalar, and structural invariance could be assumed across genders in three subjects, as evidenced by a non-significant drop in model fit (ΔCFI > −0.01) for the successively stricter models.

**TABLE 6:** Model fit statistics for models representing different degrees of invariance across genders (*N* = 1,780).

**Model**	**χ*^2^***	***df***	**Δχ*^2^***	**Δ*df***	**CFI**	**TLI**	**SRMR**	**RMSEA (90%CI)**	**ΔCFI**
**Math**									
Boys	1902.956	370	—	—	0.910	0.901	0.043	0.068 (0.065, 0.071)	
Girls	1716.869	370	—	—	0.915	0.907	0.042	0.064 (0.061, 0.067)	
Configural invariance	3630.968	741	—	—	0.912	0.904	0.044	0.066 (0.064, 0.068)	
Metric invariance	3657.092	765	26.124	24	0.912	0.906	0.046	0.065 (0.063, 0.067)	0.000
Scalar invariance	3731.893	790	74.801^∗∗∗^	25	0.910	0.908	0.047	0.065 (0.063, 0.067)	–0.002
Structural invariance	3755.034	800	23.141^∗^	10	0.910	0.909	0.053	0.064 (0.062, 0.067)	0.000
**English**									
Boys	1920.749	371	—	—	0.914	0.906	0.041	0.069 (0.066, 0.072)	
Girls	1470.427	371	—	—	0.931	0.924	0.038	0.058 (0.055, 0.061)	
Configural invariance	3391.443	743	—	—	0.922	0.915	0.040	0.063 (0.061, 0.065)	
Metric invariance	3425.393	767	33.950	24	0.922	0.917	0.042	0.062 (0.060, 0.065)	0.000
Scalar invariance	3454.017	792	28.624	25	0.921	0.920	0.043	0.061 (0.059, 0.064)	–0.001
Structural invariance	3508.579	802	54.562^∗∗∗^	10	0.920	0.919	0.081	0.062 (0.059, 0.064)	–0.001
**Chinese**									
Boys	1690.819	366	—	—	0.910	0.900	0.045	0.064 (0.061, 0.067)	
Girls	1691.285	371	—	—	0.913	0.904	0.041	0.063 (0.060, 0.066)	
Configural invariance	3382.331	738	—	—	0.911	0.902	0.043	0.063 (0.061, 0.066)	
Metric invariance	3415.148	762	32.817	24	0.911	0.905	0.045	0.063 (0.060, 0.065)	0.000
Scalar invariance	3488.201	787	73.053^∗∗∗^	25	0.909	0.907	0.047	0.062 (0.060, 0.064)	–0.002
Structural invariance	3511.186	797	22.985^∗^	10	0.909	0.907	0.055	0.062 (0.060, 0.064)	0.000

*^∗^*p* < 0.05, ^∗∗∗^*p* < 0.001.*

### Tests of Measurement Invariance Across Grades

We also tested measurement invariance across grade levels (Grades 7, 8, and 9) in sample 3. Analysis program was the same as previous. As shown in Table 7, the test of the configural invariance model with no further constraints was supported in three subjects, for math, χ^2^/*df* = 3.468, CFI = 0.918, TLI = 0.909, SRMR = 0.043, RMSEA = 0.064 (0.062, 0.067); for English, χ^2^/*df* = 3.479, CFI = 0.920, TLI = 0.912, SRMR = 0.041, RMSEA = 0.065 (0.062, 0.067); for Chinese, χ^2^/*df* = 3.487, CFI = 0.910, TLI = 0.900, SRMR = 0.045, RMSEA = 0.065 (0.063, 0.067). Metric, scalar and structural invariance could be held across grades in three subjects, as evidenced by a non-significant drop in model fit (ΔCFI > −0.01) for the successively stricter models.

**TABLE 7:** Model fit statistics for models representing different degrees of invariance across grades (*N* = 1,780).

**Model**	**χ*^2^***	***df***	**Δχ*^2^***	**Δ*df***	**CFI**	**TLI**	**SRMR**	**RMSEA (90%CI)**	**ΔCFI**
**Math**									
Grade 7	1267.097	371	—	—	0.930	0.923	0.037	0.058 (0.055, 0.062)	
Grade 8	1381.649	366	—	—	0.910	0.900	0.046	0.066 (0.062, 0.069)	
Grade 9	1152.385	359	—	—	0.911	0.900	0.046	0.072 (0.067, 0.076)	
Configural invariance	3801.130	1096	—	—	0.918	0.909	0.043	0.064 (0.062, 0.067)	
Metric invariance	3883.162	1146	82.032^∗∗^	50	0.917	0.912	0.049	0.063 (0.061, 0.066)	–0.001
Scalar invariance	3953.980	1196	70.818^∗^	50	0.916	0.915	0.050	0.062 (0.060, 0.065	–0.001
Structural invariance	4035.989	1216	82.009^∗∗∗^	20	0.915	0.914	0.066	0.063 (0.060, 0.065)	–0.001
**English**									
Grade 7	1294.899	371	—	—	0.928	0.921	0.038	0.059 (0.056, 0.063)	
Grade 8	1365.456	371	—	—	0.919	0.911	0.040	0.065 (0.061, 0.068)	
Grade 9	1204.701	369	—	—	0.910	0.901	0.046	0.073 (0.068, 0.077)	
Configural invariance	3865.057	1111	—	—	0.920	0.912	0.041	0.065 (0.062, 0.067)	
Metric invariance	3918.209	1161	53.152	50	0.920	0.916	0.045	0.063 (0.061, 0.065)	0.000
Scalar invariance	3989.891	1211	71.682^∗^	50	0.919	0.919	0.047	0.062 (0.060, 0.064)	–0.001
Structural invariance	4035.464	1231	45.573^∗∗^	20	0.919	0.920	0.062	0.062 (0.060, 0.064)	0.000
**Chinese**									
Grade 7	1380.599	371	—	—	0.910	0.902	0.043	0.062 (0.058, 0.065)	
Grade 8	1288.162	369	—	—	0.916	0.907	0.043	0.062 (0.059, 0.066)	
Grade 9	1170.025	361	—	—	0.901	0.888	0.049	0.072 (0.068, 0.077)	
Configural invariance	3838.785	1101	—	—	0.910	0.900	0.045	0.065 (0.063, 0.067)	
Metric invariance	3893.812	1151	55.027	50	0.910	0.904	0.049	0.063 (0.061, 0.066)	0.000
Scalar invariance	3958.217	1201	64.405	50	0.909	0.908	0.050	0.062 (0.060, 0.064)	–0.001
Structural invariance	4033.748	1221	75.531^∗∗∗^	20	0.907	0.908	0.062	0.062 (0.060, 0.064)	–0.002

*^∗^*p* < 0.05, ^∗∗^*p* < 0.01, and ^∗∗∗^*p* < 0.001.*

## Discussion

The current study developed and validated the Academic Interest Scale for adolescents (AISA), a new instrument for academic interest with more than 2,300 adolescents in the Chinese education context. The final scale contains 29 items and four factors, that is, *emotion, value, knowledge*, and *engagement*. This scale can generically be used for diverse subjects across different educational settings.

Hidi and Renninger’s four-phase interest development model is the most complete extant model based on the existing interest literature. Some researchers in the U.S. have developed math interest measures for elementary students according to this model (e.g., Wininger et al., 2014). Four-factor construct was obtained. Previous studies in China have developed two subject-specific interest scales (MLIQ, Wu and Liu, 2017; SLIS, Lin and Chai, 2017) for elementary and middle school students based on this model as well. But it is worth bearing in mind that the scales applied different phases as subscales instead of exploring the essence of interest. The AISA is different from the existing instruments in that it was (1) devised based on the structure and content of academic interest and the four-phase interest development model and (2) suitable for different subjects for Chinese adolescents.

Expert feedback and cognitive interview were used to initially select and modify the items in the item generation phase. EFA was conducted on the subject of math in sample 1 to refine the scale and determine factorial structure. Using a large sample (*N* = 552), we dropped more than half (52 items) of the initial items because of poor factor loading and the item discrimination index. Meanwhile, CFAs were conducted on math, English, and Chinese in sample 3 (*N* = 1,780) to further test the model fit of a 29-item scale. All indices except the χ*^2^/df* met the recommended thresholds for an adequate fit. The high value of the χ*^2^/df* was likely related to the large sample size. These results suggested that a four-factor model can appropriately capture the complex structure of academic interest in math, English, and Chinese subject in Chinese adolescents. Among these four factors, *emotion* means the extent to which a student has positive emotional response to targeted academic subject, such as liking, excitement and enjoyment. *Value* means the degree to which a student thinks that learning targeted subject is important, meaningful or useful. *Knowledge* means the level of stored knowledge for related-subject a student perceived. *Engagement* means the extent to which a student engages and reengages specific academic activities, events, and ideas over time. These findings extend prior studies which manifested four-factor structure of academic interest in elementary students in the U.S. (e.g., Wininger et al., 2014), by showing the similar structure in junior high school students in China. More broadly, these findings support the cross-culture compatibility of the four-phase interest development model.

Meanwhile, the scale scores exhibited satisfactory psychometric properties in terms of internal reliability and test-criterion relationships in math and English subjects. The composite reliability and the Cronbach’s α for the total AISA and four subscales were well above the criterion (>0.70) for adequate homogeneity (Nunnally and Bernstein, 1994). Academic interest is recognized as an important source of intrinsic motivation and flow state in learning among students (Deci, 2010; Shernoff et al., 2014). In this study, the AISA scores showed significant associations with intrinsic motivation and flow state in learning, indicating acceptable test-criterion relationships. The AISA scales had relatively poorer test-criterion relationships with flow state in learning than with intrinsic motivation. This discrepancy is probably because academic interest shares more similarity with intrinsic motivation. More suitable criterion measures should be employed in future research.

This study aimed at developing an academic interest scale suitable for interest comparison in different educational contexts, such as for different school subjects and measure occasions, with different student populations. To this end, we created the items that did not describe the unique content of a specific subject and dropped unexpected items when we conducted the EFA on math. However, proving that the functions of scale are the same in different measuring situations and the measures can be used for mean comparison were not enough (Vandenberg and Lance, 2000). In this study, we also confirmed the metric, scalar, and structural invariance of four-factor model of AISA across subjects (math, English, and Chinese), time, genders, and grade levels. Given that chi-square square difference (Δχ*^2^*) test is sensitive to sample size, we inferred measurement invariance across groups mainly based on CFI difference (ΔCFI) (Cheung and Rensvold, 2002). This approach provided stronger evidence for commonality of the AISA across subjects and proved the comparability across academic interest in math, English and Chinese, in boys and girls, in different grade levels and in different measures of time more strictly. Measurement invariances also strengthened the validity of the AISA, which implied that differences in observed test scores in different education settings could reflect true differences in academic interest rather than an artifact of the measurement method.

This study has some limitations. Our sample, adolescents from Chinese junior high schools, may limit the generalizability of the findings. Further studies should test the four-factor interest model of the AISA using larger and multiple samples, including younger children, older adolescents or even the group of adult students. Meanwhile, future studies should examine the factorial invariance of the AISA across different racial/ethnic and language groups. Another possible limitation is that correlated samples (i.e., data of math, English, and Chinese came from the same group students) were used to test the measurement invariance across different subjects. This approach may overestimate the interdisciplinary invariance of the AISA. Independent samples should be used in further test of invariance across academic subjects. The AISA was only administered for math, English, and Chinese in this study. In the future, more diverse domains (e.g., history, biology, physics, or chemistry et al.) should be included in the test of invariance across subjects.

## Data Availability Statement

The raw data supporting the conclusions of this manuscript will be made available by the authors, without undue reservation, to any qualified researcher.

## Ethics Statement

The studies involving human participants were reviewed and approved by the Ethics Committee of School of Psychology, Capital Normal University. Written informed consent from the participants’ legal guardian/next of kin was not required to participate in this study in accordance with the national legislation and the institutional requirements.

## Author Contributions

ZL conceived the research idea, made the research design, organized the research, interpreted the results, and drafted and revised the manuscript. YD conceived the research idea, made the research design, performed the studies, and analyzed the data. WX performed the studies, analyzed the data, and interpreted the results. All authors contributed to writing sections of the manuscript, and read and approved the submitted version.

## Conflict of Interest

The authors declare that the research was conducted in the absence of any commercial or financial relationships that could be construed as a potential conflict of interest.

## Appendix

**Table:** The 29-item Academic Interest Scale for Adolescents (AISA) in English and Chinese.

**Emotion**	
3	I understand the fun of
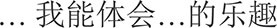
5	Studying … makes me feel happy
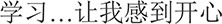
8	I am interested in
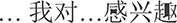
13	The content I learn from … courses is interesting
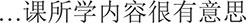
14	I enjoy studying …
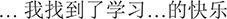
18	I really like … courses
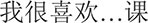
26	I enjoy when I study
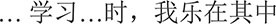
**Value**	
1	The knowledge of … is important
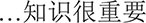
10	A good mark in … courses means a lot to me
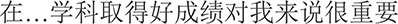
16	I think that … is helpful for my career in the future
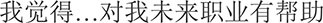
17	The knowledge of … makes my daily life easier
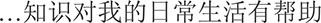
20	The knowledge of … promotes my growth
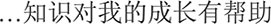
24	I find that the knowledge of … is useful in daily life
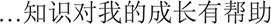
27	The knowledge of … is valuable for my future development
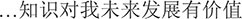
29	I think that learning … is significant for my growth
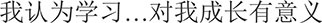
**Knowledge**	
4	I know all kinds of things about …
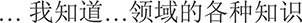
7	I am expert in
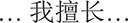
9	I can answer all kinds of questions that teachers ask in the … class
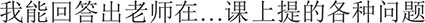
12	I am familiar with the knowledge and skills required in
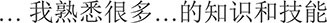
15	I do well in … lessons
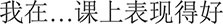
22	I have a lot of things to say about … topics
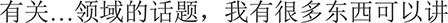
23	I have a lot of knowledge about
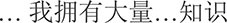
**Engagement**	
2	I want to learn things that are not included in … textbooks
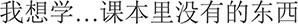
6	I hope to explore things about …
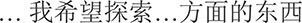
11	I will read more books about … if I have the chance
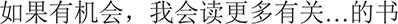
19	I want to know more things about the field of
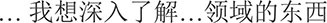
21	I will take part in an extracurricular training class for … if I have the opportunity
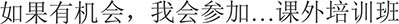
25	I want to find various ways to complete the … assignment
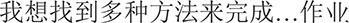
28	I am willing to spend time on the skills or methods learned from … lessons
